# Case report: Early-onset renal failure as presenting sign of Lesch-Nyhan disease in infancy

**DOI:** 10.3389/fped.2022.1080486

**Published:** 2022-12-19

**Authors:** Lianlian Yang, Hui Guo

**Affiliations:** ^1^ Department of Paediatrics, West China Second University Hospital, Sichuan University, Chengdu, China; ^2^ Key Laboratory of Birth Defects and Related Diseases of Women and Children, Ministry of Education, Sichuan University, Chengdu, China

**Keywords:** hyperuricemia, *HPRT1* gene mutation, genetic testing, congenital metabolic diseases, Lesch-Nyhan disease

## Abstract

Lesch–Nyhan disease (LND) is a rare X-linked recessive disease caused by pathogenic mutations of the HPRT1 gene. The typical clinical manifestations include cerebral palsy, intellectual disability, dysarthria, self-injurious behavior, and gouty arthritis in children. This report describes a Chinese boy aged 2 months and 7 days with a significantly elevated uric acid concentration accompanied by renal dysfunction and, notably, brain imaging changes. Whole-exome sequencing revealed a hemizygous mutation of *HPRT1* in nucleotide 508 from cytosine to thymine (c.508C > T), resulting in a nonsense mutation (p.R170X). The incidence of LND is extremely low in China, and hyperuricemia is a common clinical manifestation. Therefore, the possibility of LND should be considered in children with increased uric acid in infancy accompanied by brain imaging changes or neurological dysfunction. Moreover, genetic testing is needed to provide adequate genetic counseling to the family, and should be conducted as early as possible in such children to avoid misdiagnosis or delayed diagnosis.

## Introduction

Lesch–Nyhan disaese (LND, OMIM#300322) is an X-linked recessive disease involving mutations of the gene encoding hypoxanthine-guanine phosphoribosyltransferase (HPRT, EC 2.4.2.8). Pathogenic mutations of *HPRT1* lead to deficiency of the HPRT enzyme, resulting in dysfunction in the salvage pathway of purine nucleotide synthesis (1). The role of HPRT is to catalyze the conversion of hypoxanthine nucleoside to guanine nucleoside (2). Therefore, the deficiency of the HPRT enzyme can block such conversion *in vivo*, resulting in increased conversion of hypoxanthine nucleoside to uric acid and the development of severe hyperuricemia (3). Long-term elevation of uric acid can lead to gouty arthritis and renal impairment (4). The main synthesis pathway of purine nucleotides in humans is *de novo* synthesis, but the enzyme system involved in *de novo* synthesis of purine nucleotides is lacking in some tissues and organs, such as the brain and bone marrow; only salvage synthesis occurs at these sites. Thus, the deficiency of the HPRT enzyme may affect the development of the nervous system (5, 6). The typical clinical manifestations of LND include cerebral palsy, intellectual disability, dysarthria, self-injurious behavior, and gouty arthritis in children (1). LND is a rare disease, and the reported cases are mainly distributed in the European population. Recently, Li et al. reported the genotypes and phenotypes of LND in eight Chinese patients. The authors pointed out that the diagnosis of LND was often delayed until the emergence of self-injurious behavior, while the hyperuricaemia of individual patients was ignored. The median age of diagnosis was 31 months, and the initial manifestations were mainly head control weakness and motor retardation (7). We herein describe a Chinese boy aged 2 months and 7 days with a significantly elevated uric acid concentration accompanied by renal dysfunction and abnormal brain development. Whole-exome sequencing revealed a *de novo* mutation c.508C > T in exon 7 of *HPRT1*, resulting in a nonsense mutation of amino acid (p.R170X). The whole-exome sequencing results in both parents were normal.

## Clinical information

**Figure 1:**
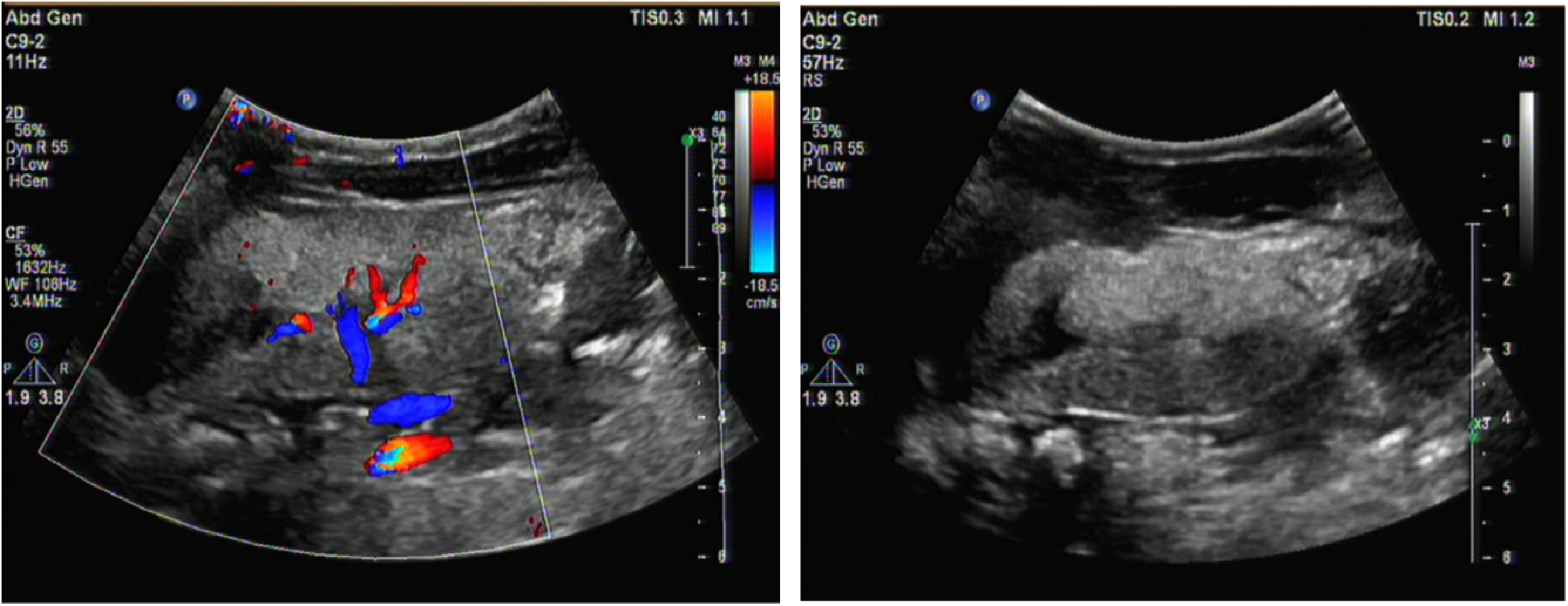
Renal ultrasound image. It revealed enhanced echoes of the parenchymaand, and unclear boundary between medulla and cortex of the bilateral kidneys as well as enlargement of both kidneys.

**Figure 2:**
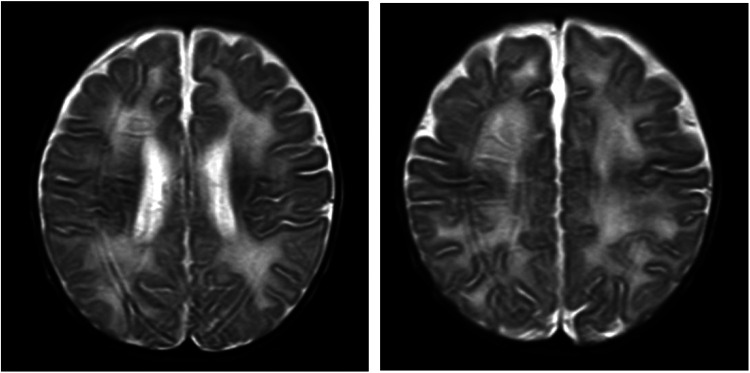
Brain MRI. Axial T2-weighted image showing diffuse white matter hyperintensity.

A male infant aged 2 months and 7 days was admitted to our hospital because of feeding difficulties, vomiting, and excessive crying at night that started around the age of 1 month. The boy was the first child of healthy and consanguineous parents (the degree of relation was the third generation) from the Zang ethnic minority group, Sichuan Province, China. He had a birth weight of 2,250 g (<P_5_), birth length 48 cm (P_10_–P_25_), and head circumference 34 cm (P_25_–P_50_). He was delivered naturally at full term without intrauterine distress or post-birth asphyxia. Physical examination on admission revealed good mental reactions, loud crying, normal muscle strength and tension of the limbs and good primary reflexes, yet masses with a diameter of about 1 cm × 1 cm were palpable on the back of both hands, the dorsum of the right feet, and the left palm. The masses exhibited moderate hardness, moderate mobility, and no increase in skin temperature. The child noisily cried when the masses were pressed. Slight swelling of the left middle finger was present. Based on those initial clinical manifestations, we expected that the patient might have gout nodules. Therefore, his uric acid concentrations were checked after admission, which were found to be significantly increased with a range from 1,192 to 1,535 μmol/L (reference range: 220–547 μmol/L) in multiple examinations; his creatinine concentrations were also elevated, fluctuating between 386 and 1,046 μmol/L (reference range: 17.3–54.6 μmol/L). Blood gas analysis revealed metabolic acidosis combined with respiratory alkalosis. Acid-base parameters and electrolytes in the blood on admission showed pH 7.28 (reference range: 7.35–7.45), sodium concentration 130 mmol/L (reference range: 135–145 mmol/L), potassium 5.1 mmol/L (reference range: 3.5–5.5 mmol/L), calcium 2.34 mmol/L (reference range: 2.25–2.67 mmol/L), phosphate 2.42 mmol/L (reference range: 1.45–2.1 mmol/L). Uric acid on admission was 1,192 µmol/L (reference range: 220–547 μmol/L), uric acid: creatinine ratio was 4.84. Urinalysis revealed that glucose, lactic acid, and ketones were all positive. Acylcarnitine and amino acid profile in the dried blood spot revealed increased C5DC, C18-OH, C3/C4 and C5DC/C8 and decreased valine and C0. Considering the findings in urine and metabolic acidosis, we judged that our patient had tubulopathy. We speculated that the decreased concentration of free carnitine was probably secondary due to tubulopathy, and the increased glutarylcarnitine was probably secondary to renal failure. Ultrasound examination of the urinary system on admission revealed enhanced echoes of the parenchyma, and unclear boundary between medulla and cortex of the bilateral kidneys, as well as enlargement of both kidneys (as shown in Figure 1). Brain magnetic resonance imaging (MRI) showed reduced T1-weighted imaging (T1WI) and fluid-attenuated inversion recovery (FLAIR) signals and increased T2-weighted imaging (T2WI) signals in the bilateral cerebral hemispheres and in the frontal, parietal, temporal, and occipital white matter areas, suggesting an increased white matter water content (as shown in Figure 2). Multiple subcutaneous masses in the flexion area of the right upper arm and left palm were found to be gout nodules by ultrasound. Whole-exome sequencing of DNA in the peripheral blood of the child and his parents revealed one hemizygous mutation in the *HPRT1* gene of the child [c.508C > T (p.R170X)]. It is worth noting that the mother didn't harbor the mutation. Therefore, the mutation in the child was *de novo*. The mutation was nonsense, and identified as a pathogenic mutation according to the guideline from the American College of Medical Genetics and Genomics. After admission, the patient was given oral sodium bicarbonate to correct the acid–base imbalance and was treated with allopurinol (2.6 mg/kg/d) for 2 weeks to reduce the uric acid concentration. After 2 weeks of treatment, the acid-base parameters and electrolytes in the blood of our patient returned to normal levels. Uric acid and creatinine concentrations were significantly lower than those on admission, but still at high levels. His uric acid concentration was decreased to 782 μmol/L (reference range: 220–547 μmol/L), and creatinine concentration was 82.5 (reference range: 17.3–54.6 μmol/L). Two weeks after the treatment in hospital, the parents voluntarily requested discharge of the child from hospital. We informed the parents that the patient should continue taking allopurinol after discharge, and recheck the concentrations of uric acid and creatinine 1 week later. Follow-ups showed that concentrations of uric acid and creatinine of the child returned to normal levels 1 week later, whereas the child manifested head control weakness and could not reach for objects at 4 months old, could not roll over or hold objects with both hands at 6 months old, and could not sit unsupported at the age of 8 months.

## Discussion

To the best of our knowledge, the age of our patient on hospital admission was the youngest among the LND patients reported from mainland China so far (7–9). The patient at admission had somewhat atypical presentations of LND with significantly elevated uric acid concentrations accompanied by renal dysfunction and multiple gout nodules. Yet notably, our MRI examination of the young infant revealed brain imaging changes.

LND is a rare single-gene disorder related to uric acid metabolism. It is associated with pathogenic mutations in *HPRT1* gene and can be classified into three clinical manifestations with different severities according to differences in the degree of reduced HPRT enzyme activity (10–12):

• typical LND: HPRT enzyme activity of <1.5%, gout, nervous system dysfunction, self-injurious tendency, and early onset with rapid progression to complete loss of HPRT enzyme activity
• intermediate type: HPRT enzyme activity of 1.5%–8.0%, gout, and neurological dysfunction without self-injurious tendency
• mild type: HPRT enzyme activity of >8.0%, only hyperuricemia (3)

All three types are related to the overproduction of uric acid. However, it has been reported that patients who were diagnosed with LND before birth and started taking allopurinol immediately after birth without hyperuricemia still had self-injurious behavior, suggesting that the self-injurious behavior is not caused by hyperuricemia or excessive hypoxanthine (13). Thus, the neurological dysfunction pathogenesis remains unclear. Our patient on hospital admission was only 2 months and 7 days old. We cannot predict whether he will have manifestations of the self-injurious tendency or neurological dysfunction in future (such as arrested development, low systemic muscle tension, athetosis or dance-like involuntary movement, restlessness, slurred speech, or convulsions). Of note, the child had excessive crying at night and the proneness to milk-vomiting, which might be the early manifestations of the disease.

Children with LND usually develop neurological dysfunction from 3 to 6 months after birth. The initial manifestations are developmental delay, low muscular tension, and the inability to sit without support. Later, extrapyramidal and pyramidal symptoms gradually appear. The extrapyramidal symptoms include dystonia, athetosis, and opisthotonus, and the pyramidal symptoms include tendon hyperreflexia and spastic paralysis. Affected children usually develop behavioral abnormalities and cognitive impairment at 1–3 years of age, possibly with microcephaly and seizures. Of the behavioral abnormalities, self-injurious behavior is a relatively typical manifestation and can present as self-biting of the lips, tongue, and fingers; hitting of the head and limbs; and, in severe cases, truncation of finger joints and loss of teeth (5–7). In the present case, the child was only 2 months and 7 days old and showed no obvious mental or motor disabilities during the physical examination on admission.

Declined HPRT enzyme activity affects the synthesis of purine nucleotides in the brain, and brain imaging often reveals nonspecific brain atrophy. In this case, brain MRI showed reduced T1WI and FLAIR signals and increased T2WI signals in the bilateral cerebral hemispheres and in the frontal, parietal, temporal, and occipital white matter areas. These changes suggested an increased white matter water content, consistent with early onset brain atrophy. The declined HPRT enzyme activity blocks conversion of the hypoxanthine nucleoside into the guanine nucleoside *in vivo* and then causes conversion of the hypoxanthine nucleoside into uric acid *in vivo*, which leads to elevated uric acid in the blood and urine and deposition of uric acid crystals in the kidneys, ureters, and bladder. Urinary tract ultrasound can indicate the most severely affected regions of the renal collecting system. In this case, multiple urological ultrasound examinations showed enhanced parenchymal echoes and enlarged kidneys. If the disease cannot be detected early, the continuous elevation of uric acid will result in gouty arthritis and gout nodules. At hospital admission, our patient presented multiple masses in his right upper arm, left forearm, left elbow, left middle finger and back of the left hand, bilateral dorsum of the feet, and the right leg. Based on the combined presence of these masses with the continuously high blood uric acid levels, the patient was considered to have gout nodules. Diagnosis of LND requires detection of HPRT enzyme activity and *HPRT1* gene mutation (10). In this case, whole-exome sequencing indicated a mutation of c.508C > T in exon 7 of the *HPRT1* gene, causing a nonsense mutation of amino acid (p.R170X). The whole-exome sequencing results in both parents were normal, so the mutant line was newly generated.

At such a young age of 2 months, the child in this case had multiple systemic gout nodules, renal dysfunction, and significantly increased uric acid concentrations in multiple examinations. It is easy for clinicians to simply consider uric acid nephropathy but ignore the underlying cause of the high uric acid, resulting in misdiagnosis or delayed diagnosis. Our patient was diagnosed early through genetic testing before the onset of typical neurological signs and self-injurious behavior. Therefore, uric acid, as a biomarker for purine metabolism disorders, should be tested in children with renal stones, nephrocalcinosis, renal dysfunction, neurological problems, or brain imaging changes (14). In particular, for male patients with elevated uric acid in infancy accompanied by brain imaging changes, intellectual disability or neurological dysfunction, clinicians should be highly alert to the possibility of LND, and genetic testing should be conducted as early as possible.

## Data Availability

The original contributions presented in the study are included in the article/Supplementary Material, further inquiries can be directed to the corresponding author.

## References

[1] Bell S Kolobova I Craper L Ernst C. Lesch-Nyhan syndrome: models, theories, and therapies. Mol Syndromol. (2016) 7:302–11. 10.1159/000449296 27920633 PMC5131334

[2] Jinnah HA De GL Harris JC Nyhan WL O'Neill JP. The spectrum of inherited mutations causing HPRT deficiency: 75 new cases and a review of 196 previously reported cases. MutatRes. (2000) 463:309–26. 10.1016/S1383-5742(00)00052-1 11018746

[3] Keehaugh AC Sullivan RT Thomas JW. Gene duplication and inactivation in the HPRT gene family. Genomics. (2007) 89:134–42. 10.1016/j.ygeno.2006.07.003 16928426

[4] Torres RJ Puig JG. Hypoxanthine—guanine phosophoribosyltransferase (HPRT) deficiency: lesch—nyhansyndrome. Orphanet J Rare Dis. (2007) 2:48. 10.1186/1750-1172-2-48 18067674 PMC2234399

[5] Jinnah HA Visse JE Haris JC Verdu A Larovere L Ceballos-Picot I Delineation of the motor disorder of lesch—nyhan disease. Brain. (2006) 129:1201–17. 10.1093/brain/awl056 16549399 PMC3508431

[6] Göttle M Prudente CN Fu R Sutcliffe D Pang H Cooper D Loss of dopamine phenotype among midbrain neurons in lesch—nyhan disease. Ann Neurol. (2014) 76:95–107. 10.1002/ana.24191 24891139 PMC4827147

[7] Li L Qiao X Liu F Wang J Shen H Fu H Description of the molecular and phenotypic spectrum of Lesch-Nyhan disease in eight Chinese patients. Front Genet. (2022) 13:868942. 10.3389/fgene.2022.868942 35559039 PMC9086273

[8] Huang J Zhang C Guo Q Zhang X Ma L Zhan Y Lesch-Nyhan syndrome in a Chinese family with mutation in the hypoxanthine-guanine phosphoribosyltransferase gene. Clin Lab. (2018) 1(64):197–200. 10.7754/Clin.Lab.2017.170813 29479880

[9] Jian WX Peng WH Li HL Feng QW Wang WX Su Q. Molecular characterization and structure analysis of HPRT in a Chinese patient with Lesch-Nyhan disease. Nucleos Nucleot Nucl. (2013) 32:189–95. 10.1080/15257770.2013.774013 24001192

[10] Harris JC. Lesch-Nyhan syndrome and its variants: examining the behavioral and neurocognitive phenotype. Curr Opin Psychiatry. (2018) 31:96–102. 10.1097/YCO.0000000000000388 29227296

[11] Fu R Sutcliffe D Zhao H Huang X Schretlen DJ Benkovic S Clinical severity in Lesch-Nyhan disease the role of residual enzyme and compensatory pathways. Mol Genet Metab. (2015) 114:55–61. 10.1016/j.ymgme.2014.11.001 25481104 PMC4277921

[12] Fu R Chen CJ Jinnah HA. Genotypic and phenotypic spectrum in attenuated variants of Lesch-Nyhan disease. Mol Genet Metab. (2014) 112:280–5. 10.1016/j.ymgme.2014.05.012 24930028 PMC4122630

[13] Jinnah HA. Lesch nyhan disease from mechanism to model and back again. Dis Model Meeh. (2009) 2:116–21. 10.1242/dmm.002543 PMC2650214 19259384

[14] Jasinge E Kularatnam GAM Dilanthi HW Vidanapathirana DM Stiburkova B. Uric acid, an important screening tool to detect inborn errors of metabolism: a case series. BMC Res Notes. (2017) 10:454. 10.1186/s13104-017-2795-2 28877755 PMC5588617

